# From the London International Cough Symposium to the ERS Cough Conference: chronic cough comes of age

**DOI:** 10.1183/23120541.00951-2026

**Published:** 2026-07-27

**Authors:** Kian Fan Chung

**Affiliations:** 1 National Heart and Lung Institute, Imperial College London, London, UK; 2 Royal Brompton and Harefield Hospitals, Guy's and St Thomas’ NHS Foundation Trust, London, UK

## Abstract

The first European Respiratory Society (ERS) Cough Conference was held in London at the Royal College of Physicians in London on 17–19 July 2026 (https://channel.ersnet.org/event-390-ers-cough-conference-2026). This event marked the emergence of chronic cough not only as a symptom but as a disease of its own, which is reflected in the main theme of the Conference, “Chronic cough as a disease”.

## Shareable abstract

**The @EuroRespSoc Cough Conference** https://bit.ly/4asS7NF

The first European Respiratory Society (ERS) Cough Conference was held in London at the Royal College of Physicians in London on 17–19 July 2026 (https://channel.ersnet.org/event-390-ers-cough-conference-2026). This event marked the emergence of chronic cough not only as a symptom but as a disease of its own, which is reflected in the main theme of the Conference, “Chronic cough as a disease”.

This Conference represents the culmination of efforts over the past 50 years since the publication of the definition of chronic cough on the basis of its duration of >8 weeks and the clinical approach to the management of patients with chronic cough in 1981 [1]. At that time, cough was increasingly being recognised as one of the most common symptoms for which people present to primary care and as a chief complaint of patients seeking medical attention in respiratory or allergy specialist clinics.

Then came the realisation that a high proportion of these people presenting with a chronic cough did not have any underlying condition associated with the cough (unexplained or idiopathic chronic cough) and also those in whom treatment of any underlying condition linked to the cough had no effect on the cough (refractory chronic cough) [2]. This started the inquisition into the basis for the persistence and refractoriness of chronic cough.

## The London International Cough Symposium: 1996–2024

This endeavour was catalysed by the setting up of symposia focusing on chronic cough, the first one ever being the London International Cough Symposium (LICS) initiated by John Widdicombe in October 1996. The LICSs, all held at the National Heart and Lung Institute, Imperial College London, were biennial events with the Thirteenth Symposium, the last, held in 2024, gathering scientists, clinicians and those involved in the care of chronic cough patients in understanding better the pathophysiology and clinical aspects of chronic cough.

John Widdicombe was one of the first to characterise Aδ-myelinated fibres in the airways that could mediate cough and increased breathing [3]. Under his guidance, our understanding of chronic cough, both at the pathophysiological and clinical levels, blossomed. While the initial symposia focused on cough as a symptom and its definition, its modulation peripherally by airway reflexes and at a central cortical level, later symposia focused on the neural activation pathways related to the network components that control voluntary cough, cough suppression and the urge to cough.

The sixth LICS in 2010 discussed how the cough centre can be tuned and it was from this Symposium that the concept of cough hypersensitivity syndrome (CHS) was conceived, which translated the concept of cough as a neuropathic process to the clinic [4]. For the eighth LICS held in July 2014, the focus was devoted to this novel concept of CHS as forming the basis for chronic cough [5].

Cough hypersensitivity was characterised by increased cough responses to stimuli that affect the airways and vagally innervated tissues as well as by excessive cough responses to innocuous stimuli [6, 7]. There was the recognition that it was caused by neuroinflammatory and neuropathic mechanisms at both the peripheral and central levels, which laid the foundation for the use of centrally acting neuromodulators as antitussives, and later on for the development of P2X3 blockers as a peripheral antitussive that is now available in the clinic [4, 8]. The later symposia very much focused on the pharmacological and therapeutic aspects on chronic cough with presentation of all ongoing clinical studies and trials in that field (table 1). Cough hypersensitivity with laryngeal hypersensitivity has now been put into application in the ERS guidelines on the diagnosis and treatment of chronic cough in adults and children [20].

**TABLE 1:** The London International Cough Symposium (LICS) (1996–2024)

LICS	Year	Theme	Refs
**1st**	1996	Methods and mechanisms	[9]
**2nd**	2001	Pharmacology and therapy	[10]
**3rd**	2004	Cough as a symptom: acute and chronic	[11]
**4th**	2006	Assessment and measurement of cough	[12]
**5th**	2008	Mechanisms and treatment	[13]
**6th**	2010	Cough: a translational approach	[14]
**7th**	2012	Cough: hypersensitivity and allosterocity In memoriam of John Widdicombe	[3, 15]
**8th**	2014	Cough hypersensitivity syndrome as basis for chronic cough	[16]
**9th**	2016	Mechanisms and management of chronic cough	[17]
**10th**	2018	New antitussive therapies	[18]
**11th**	2021	Web-based meeting (COVID-19 restrictions)	
**12th**	2022	Mechanistic insights into antitussives	[8]
**13th**	2024	Cough as a disease	[19] https://medicsevents.co.uk/events/the-thirteenth-london-international-cough-symposium-13th-lics/

Finally, the Thirteenth Symposium focused on the concept of chronic cough as a disease, delving into the implications of this concept for practice, research and healthcare [19]. In this respect, the approach to the management of this disease needs further consideration and the application of the concept of cough hypersensitivity needs further exploitation. In short, the vision of John Widdicombe by the time he passed away in 2011 was realised at that last Symposium. These symposia can be considered to be the driving catalyst of many of the recent discoveries and progress in our understanding of chronic cough as a symptom and, more recently, as a disease.

Other international meetings were later organised, with the American Cough Conference started by Peter Dicpinigaitis in June 2007 [21] and the Chinese International Cough Conference started by Kefang Lai in 2013 [22]. Both conferences will be held next year in 2027 and have also contributed to this progress.

## ERS Cough Conference 2026

With the increasing recognition that chronic cough is now a disease, it is natural that the cough conference would need to take on a larger audience. The ERS taking over the running of the LICS was a very welcome move. An Organising Committee (Kian Fan Chung, Omar Usmani, Didier Cataldo, Marta Dabrowska, Stuart Mazzone, Esther Palones, Jana Plevkova and Imran Satia) set up in August 2025 was tasked to prepare for the first ERS Cough Conference. The objectives were to: 1) deliver the latest clinical and scientific breakthroughs in cough research; 2) create a unique forum connecting patients, clinicians and scientists to exchange insights on pathophysiology and treatment; and 3) advance education on chronic cough management across all levels of healthcare, following its recognition as a distinct disease. These objectives went beyond those of previous cough symposia with the greater active participation of patients and a greater emphasis on education of cough as a disease to the medical and patient communities, as well as the general public.

There were four main sessions that reviewed the diseases associated with chronic cough, the advances in understanding of different types of cough, the underlying pathophysiological mechanisms and the management of chronic hypersensitive cough. In addition, there were sessions on discovery science and innovation in chronic cough in collaboration with industry partners, including the current evolving scene for new treatment approaches.

Central to the 3-day meeting was the John Widdicombe lecture to commemorate his legacy that was delivered by Woo-Jung Song of Asan Medical Center, University of Ulsan College of Medicine in Seoul, South Korea.

75 abstracts covering cough assessment, measuring cough and cough hypersensitivity, pathophysiology, and treatments from across Europe, the Middle East, North America, Asia and Australia were accepted, and these were presented and discussed during the plenary sessions, with some as oral presentations. These abstracts will be published as a supplement to *ERJ Open Research*. This is evidence of the increasing interest in investigating chronic cough as a disease and the need to find effective treatments and better management approaches.

## Final note

On a personal note, I was pleased that John Widdicombe asked me to join him to organise the LICS meetings after the first one held in 1996, a proposition that I did not hesitate to accept. A personal thanks to all those scientists and clinicians who have contributed to the past LICSs to make it a forum where the discussions have contributed a lot to the advancement of the field. Apart from myself, only Alyn Morice has been present at all 13 LICSs!

So, the ERS Cough Conference 2026 succeeds the LICS and I am pleased to continue my involvement after the long experience gained over the past 30 years.

Welcome to the ERS Cough Conference: this marks another stage in the history of chronic cough which is coming of age.
